# Novel Deposition Technique for Fabricating Films with Customized Thickness Profiles

**DOI:** 10.3390/mi15121412

**Published:** 2024-11-23

**Authors:** Chi-Yung Hsieh, Yu-Chi Lin, Xuan-Shan Huang, Jing-Ting Lin, Cheng-Sheng Huang

**Affiliations:** Department of Mechanical Engineering, National Yang Ming Chiao Tung University, Hsinchu 30010, Taiwan; h.c@nycu.edu.tw (C.-Y.H.); yourge067.en08@nycu.edu.tw (Y.-C.L.); owenhuang0711@gmail.com (X.-S.H.); linjt880926.en11@nycu.edu.tw (J.-T.L.)

**Keywords:** shadow mask, gradient thickness, linear variable filter, film deposition

## Abstract

This study introduces a novel deposition technique capable of depositing thin films with any arbitrary thickness profile. The apparatus consists of a fixed shadow mask and a rotating sample carrier plate. The shadow mask features a specifically designed opening curve that corresponds to the particular thickness profile of the deposited film. We successfully designed two shadow masks and used them to deposit films with linear thickness gradients of 49.3 and 86.8 Å/mm and films with sinusoidal thickness profiles with a period of 40 mm. Furthermore, a linear variable filter was designed on the basis of a quarter-wavelength stack of Si_3_N_4_ and SiO_2_, combined with a TiO_2_ cavity layer with a linearly varying thickness. By coaxially rotating the sample carrier plate relative to the shadow mask, films with the desired thickness profiles could be fabricated in a single deposition step without the need for additional rotational or translational devices inside the deposition chamber. By rotating the carrier plate, the chips attached at different circumferential positions can achieve consistent thickness profiles, making this method well-suited for mass production.

## 1. Introduction

Thickness-gradient thin films can be used in optical devices for various applications. For example, they can be used in linear variable filters (LVFs) [1] for biosensing applications [2] and spectral or hyperspectral measurements [3]. In addition, they can be used to create tunable guided-mode resonance filters [4], which have use in spectral detection or in biosensing chips [5,6]. However, to ensure that the fabricated thickness-gradient films can be widely used in commercial applications, their thickness gradient must be customizable and the fabrication method must support mass production—both of which are challenging criteria to meet.

During the early stages of its development, optical filtering was primarily achieved using colored and glass filters. Advancements in thin-film technology led to the creation of more efficient filters based on principles of thin-film interference. Traditionally, optical filters are designed to operate at a specific wavelength; to operate at a different wavelength, the instruments used require custom mechanisms to hold and switch between various filters [7].

In 1977, Theodore R. Owen patented the design of a linear wedge spectrometer [8]. Using this instrument, the filtering wavelength could be precisely controlled by varying the optical film thickness. This innovation, which can be applied in spectrometers or filters, enabled the design of a single-chip spectrometer [9].

The fabrication of thickness-gradient films requires high precision to ensure that the variation in thickness is stable and uniform; even a minor defect or nonuniformity can lead to unexpected optical effects, affecting the performance of the filter. Additionally, the variation in thickness must be designed within a fixed range to balance cost-effectiveness with the feasibility of large-scale production. Complex deposition techniques, such as electron beam evaporation or sputtering, are usually used for fabricating thickness-gradient films. The ideal fabrication technique should offer both designability and scalability, so that cost-effective products tailored to the required size can be manufactured.

Several fabrication methods for thickness-gradient films have been proposed. Wang et al. (2006) proposed a method involving multiple mask alignment exposure followed by etching [10]. The same research team proposed a combinatorial deposition technique in 2007 to replace the etching process [11]. Both processes are highly complex and poorly suited to the production of multilayered thickness-gradient films. Piegari et al. (2008) manufactured a thickness-gradient film by using a stepper motor to move a square mask in one direction. The movement speed of the mask was controlled through software [12]. This method is demanding and unsuitable for the production of multiple chips in parallel because it requires special electromechanical devices inside the deposition chamber that must be connected to an external computer for control. Abel-Tiberini et al. (2008) demonstrated the manufacture of thickness-gradient films using (1) an eccentric circular mask combined with a rectangular mask and (2) a square mask driven by an eccentric cam relative to the rotating substrate [13]. This method allows for the large-scale production of overlapping multilayered thickness-gradient films; however, the designability of masks for creating films of various thickness profiles was not explored using this method. Emadi et al. (2009) used a process involving photoresist etching, high-temperature reflow, and re-etching to create thickness gradients [14], but this method is only suitable for producing monolayers. Qian et al. (2016) combined the use of an etching mask with an ion etching method to fabricate a thickness-gradient film [4]. A mask with triangular openings was used, and the sample was moved back and forth behind the etching mask to unevenly etch the film. Tang et al. (2018) engineered a hollow mask with a specially designed pattern [15] based on the mask design proposed by Abel-Tiberini [13]. The substrate was rotated along the edge of the mask’s opening to achieve a thickness-gradient film. However, aligning the center of the hollow mask with the center of the substrate is a challenge. Additionally, designing mask openings tailored to the fabrication of films with thickness profiles other than that of linear gradient thickness was not discussed.

In this study, we propose a novel method for the fabrication of thickness-gradient films that features customizable mask design and ease of mass production. The apparatus comprises a fixed shadow mask and a rotating carrier plate. The carrier plate is rotated in concentric circles, resulting in the formation of chips with consistent thickness profiles at different locations on the plate. This fabrication method is amenable to batch production. Multilayered films with the same gradient, that are composed of various materials, can be easily produced using suitable deposition machines. The proposed mask design can be used to fabricate films with any thickness profile.

## 2. Materials and Methods

**Figure 1:**
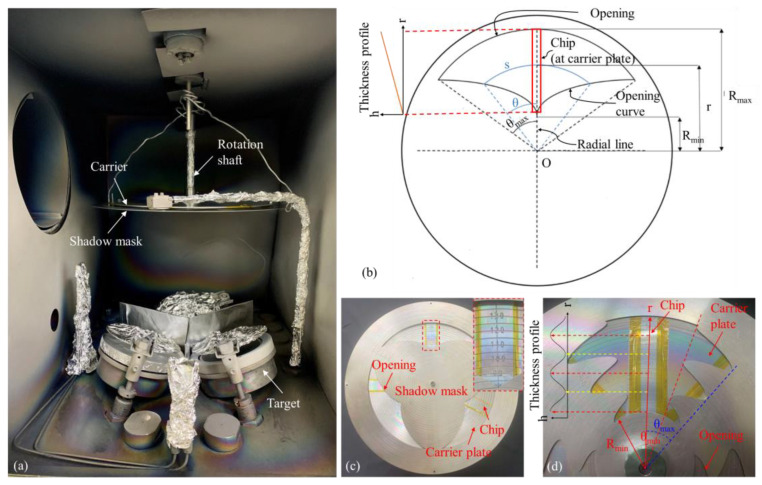
(**a**) Interior of the sputtering chamber and the apparatus used to deposit films with arbitrary thickness profiles. (**b**) Schematic of a shadow mask with a single opening. (**c**) Shadow mask and carrier plate with mounted glass chips. (**d**) Sinusoidal shadow mask exhibiting correspondence between the glass chips and the expected sinusoidal thickness profile.

The proposed method was validated using an sputtering machine (Microvac 450CB, Ion Tech, Inc., Fort Collins, CO, USA). Figure 1a displays the interior of the sputtering chamber, which was composed of three sputtering targets. Only one sputtering target was used at a time. The figure illustrates the arrangement of the shadow mask, carrier plate, and chips. Thin films with a particular thickness profile were deposited on either glass or Si chips, which served as substrates. The substrate was placed on the carrier plate, which was connected to a rotating shaft. The shadow mask was connected to the rotating shaft through a polyether ether ketone bearing and was fixed inside the chamber to ensure that it did not rotate with the carrier plate. During the deposition process, the carrier plate and the chips were rotated continuously, whereas the shadow mask remained stationary.

Figure 1b shows an example of a shadow mask with one opening, which is symmetrical with respect to the radial line. s represents the arc length at a specific radial distance r, with a corresponding arc angle of 2θ with respect to the origin. A chip to be deposited is represented as a red rectangle attached to the carrier plate. As the carrier plate rotates, the chip moves through the shadow mask opening and is exposed to the target molecules. Assuming that the target molecules are uniformly distributed in the deposition chamber, the deposition thickness (*h*) at any point on the chip is proportional to the deposition rate (*α*) and the total deposition time (*T*) when the shadow mask is not used. This relationship can be expressed as *h* = *αT*. The deposition thickness is independent of the radial position.

When the shadow mask is used (Figure 1b), the exposure time at radial position *r* during each rotation is proportional to the ratio of the arc length to the circumference, which is equivalent to the ratio of twice the arc angle 2*θ*(*r*) to 2*π* radians. The exposure time *t*(*r*) at position r is expressed as follows:

(1) $$t(r)=T\frac{\theta (r)}{\pi }$$

Therefore, the thickness at position *r* can be expressed as follows:

(2) $$h(r)=\alpha t(r)=\alpha T\frac{\theta (r)}{\pi }$$

A larger arc angle 2*θ*(*r*) at a specific radial position entails longer exposure time at that position, leading to a thicker film.

The corresponding opening curve can be designed on the basis of the desired variation in the thickness profile of the film. For example, to fabricate a film with a linear variation in thickness, the arc angle *θ*(*r*) must vary linearly along the radial direction according to the following simple linear relationship.

(3) $$\theta \left(r\right)=\frac{\theta_{max}}{R_{max}-R_{min}}\left(r-R_{min}\right)$$

where $R_{min}$ and $R_{max}$ define the boundaries of the opening, and $\theta_{max}$ is the maximum arc angle corresponding to the outer edge of the opening.

The thickness of the film at radial position *r* can then be expressed as follows:

(4) $$h\left(r\right)=\alpha t=\frac{\alpha T}{\pi }\frac{\theta_{max}}{R_{max}-R_{min}}\left(r-R_{min}\right)$$

The thickness gradient g of the deposited film can be determined from the following equation:

(5) $$g=\frac{dh}{dr}=\frac{\alpha T}{\pi }\frac{\theta_{max}}{R_{max}-R_{min}}$$

$R_{min}$ and $R_{max}$ are predetermined and can be designed using the chip size to determine the radial distance of the shadow mask opening. $\theta_{max}$ represents the maximum arc angle corresponding to the outer edge and can also be arbitrarily defined. A greater $\theta_{max}$ signifies a longer exposure duration when the carrier plate completes each revolution, resulting in a thicker film at the outer surface. For a given set of values for $R_{min}$, $R_{max}$, α, and $\theta_{max}$, the thickness gradient during film deposition can be further adjusted on the basis of the deposition time *T*, according to Equation (5).

Figure 1c shows a picture of the shadow mask and a carrier plate with attached chips fabricated by this study. This shadow mask had three identical openings to increase the exposure time of the chips with each revolution, thereby reducing the total deposition time required to achieve the desired deposition thickness. As shown in the inset, the mask and carrier plate were placed at the same radial distance from the origin. Distance markings and circumferential engravings were used to conveniently adhere the chips at the same radial position during fixation, allowing multiple chips to be simultaneously coated with films that have the same thickness profiles. Additionally, this setup provided positional correspondence for subsequent thickness or spectral measurements. The openings were cut into an aluminum plate (diameter, 380 mm; thickness, 1 mm) using a fiber laser cutting machine. Concentric circumferential lines and distance labels were engraved on the mask and carrier plate (diameter, 320 mm; thickness, 1 mm) using a computer numerical control (CNC) engraving machine; these lines and labels served as distance markers when multiple chips were placed.

In addition to manufacturing a shadow mask for depositing films with a linear thickness gradient, we designed a shadow mask for depositing films with a sinusoidal variation in thickness. This mask also contains three identical openings to reduce the deposition time. Figure 1d shows part of the mask and the glass chip attached on the carrier plate. According to Equation (3), the film thickness at a particular radial location *r* is proportional to the ratio of the arc angle to 2*π* radian. To achieve a sinusoidal variation in thickness, the arc angle must also vary sinusoidally with the radial position r according to the following equation:

(6) $$\theta \left(r\right)=\frac{\left(\theta_{max}+\theta_{min}\right)}{2}+\frac{\left(\theta_{max}-\theta_{min}\right)}{2}sin(2\pi \frac{r-R_{min}}{\tau }+\frac{\pi }{2})$$

The resulting film thickness can then be calculated using the following equation:

(7) $$h\left(r\right)=\alpha t=\frac{\alpha T}{\pi }\left[\frac{\left(\theta_{max}+\theta_{min}\right)}{2}+\frac{\left(\theta_{max}-\theta_{min}\right)}{2}sin(2\pi \frac{r-R_{min}}{\tau }+\frac{\pi }{2})\right]$$

where τ represents the intended period of the sinusoidal thickness profile, which was 40 mm in this study; $R_{min}$ can be set according to the expected starting position of the mask opening, which was 50 mm from the origin in this study; and $\theta_{max}$ and $\theta_{min}$ can be set according to the desired thickness variation. If a gradual thickness variation is desired, the values of $\theta_{max}$ and $\theta_{min}$ can be set in such a manner that the difference between the values is small; conversely, if a rapid change in thickness is required, the values of $\theta_{max}$ and $\theta_{min}$ can be set to have a large difference. In this study, $\theta_{max}$ and $\theta_{min}$ were set to 40° and 18°, respectively.

The sinusoidal thickness profile is also shown in Figure 1d. The peaks in the figure—indicated by the red dashed arrows at 50, 90, and 130 mm—correspond to the radial locations *r* with the maximum arc angle ($\theta_{max}$). Conversely, the valleys in the figure—indicated by the yellow dashed arrows at 70 and 110 mm—correspond to the radial locations with the minimum arc angle ($\theta_{min}$).

## 3. Results and Discussion

### 3.1. Linear Thickness-Gradient Film

**Figure 2:**
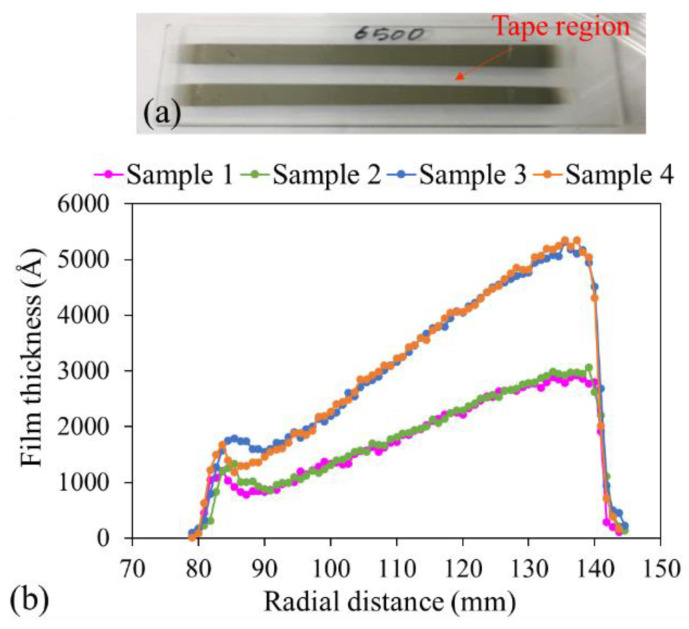
(**a**) Picture of the Ni film deposited on the glass chip with the tape region in the center. (**b**) Thickness profiles of Ni films deposited on four glass chips exhibiting two thickness gradients resulting from different deposition times using the same shadow mask.

A thin Ni film with a linearly variable thickness was deposited using a shadow mask, as shown in Figure 1c. Four glass chips (25 mm × 75 mm, referred to as samples in Figure 2b) were placed on the carrier plate at the same radial distance but at different circumferential positions, all pointing toward the origin, as shown in Figure 1c. After reaching a base pressure of 4 × 10^−6^ torr, Ar gas was introduced into the chamber at a flow rate of 24 sccm. Once the chamber pressure stabilized at 7.6 × 10^−3^ torr, the DC power was activated and set to 180 W. The sputtering deposition proceeded in two stages. The first stage was suspended when the reading of the quartz crystal monitor on the sputtering machine reached 3750 Å, and two samples were then removed. In the second stage, deposition on the remaining two samples resumed, with their position left unchanged, for an additional 2750 Å, resulting in a thicker film with a thickness of 6500 Å according to the reading on the quartz crystal monitor.

Before deposition, Kapton vacuum heat-resistant tape was applied to the center of the glass chips. After deposition, the tape was peeled off, creating a step in the Ni film, as shown in Figure 2a (tape region). A stylus profilometer (Dektak XT, Bruker Scientific Instruments, Billerica, MA, USA) was used to measure the step height of the thin film on the glass chips. To ensure the accuracy of deposition and the subsequent measurements, the edges of the tape were pressed firmly to prevent sputtering molecules from depositing underneath the tape. The exposed area had a radial distance between 80 and 140 mm (Figure 1c). Figure 2b summarizes the variation in the thicknesses of the Ni films on the four samples at different circumferential positions. Ni was chosen for its higher deposition rate and its opacity; these features facilitate the manual alignment of the profilometer probe with the step in the thin film during thickness measurements.

The thickness profiles displayed in Figure 2b empirically validate the implication of Equation (4) that the thickness gradient can be easily controlled by controlling the total exposure time. The deposition time for Samples 3 and 4 was 1.73 times that for Samples 1 and 2, assuming a constant deposition rate. Between the radial distances of 90 and 130 mm, the thickness gradient for Samples 1 and 2 was 49.3 Å/mm, whereas that for Samples 3 and 4 was 86.8 Å/mm—approximately 1.76 times the corresponding value for Samples 1 and 2. The thickness gradient g achieved using the shadow mask can be determined using Equation (5); the thickness gradient increases with the deposition time. The mask design and deposition time must be coordinated to achieve the desired thickness gradient within a fixed chip size.

The desired thickness gradient and thickness range can be flexibly achieved by adjusting the total deposition time and radial positions of the chips on the carrier plate. The effect of the nonuniform distribution of target molecules inside the chamber is minimized as a consequence of the rotation of the carrier plate and the chips during the deposition process. The experimental results indicated that the use of glass chips from the same batch placed at the same radial position but different circumferential positions can yield the same gradient and thickness within the same deposition time. As shown in Figure 2b, except for the ends of each pair of glass chips, where edge effects were prominent, approximately 80% of the central section was coated with films that had the same gradient and thickness, thus enabling batch processing.

### 3.2. Sinusoidal Film Thickness Profile

The four Si chips (A, B, C, D) were taped around the edges to the carrier plate using Kapton tape at radial distances between 50 and 150 mm (Figure 1d). A shadow mask was used for SiO_2_ deposition with a Si target, resulting in a sinusoidal thickness profile. After reaching a base pressure of 4 × 10^−6^ torr, Ar and O_2_ gases were introduced into the chamber at flow rates of 24 and 12 sccm, respectively. Once the chamber pressure reached 7.6 × 10^−3^ torr, the DC power was activated and set to 50 W. The experiment involved four rounds of deposition with identical durations. After each round, the chamber was vented to remove one Si chip, while the remaining chips were subjected to the next round of deposition. This process was repeated until all four rounds were complete, and all four chips were removed.

**Figure 3:**
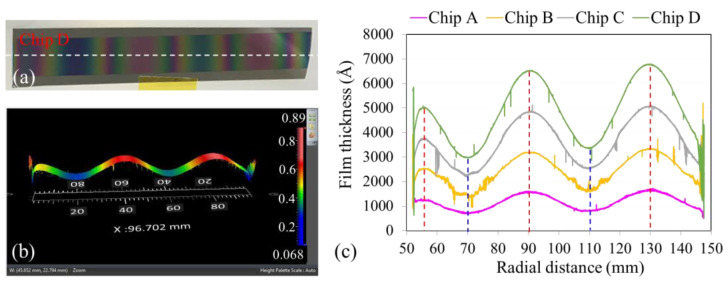
(**a**) SiO_2_ film deposited on a Si wafer using a shadow mask, as shown in Figure 1d. (**b**) Measured thickness profile. The legend represents the thickness in micrometers. (**c**) SiO_2_ thickness profile and corresponding peaks and valleys across four Si chips.

The deposition process results in the formation of colorful SiO_2_ films on Si chips. The color of the film varies according to its thickness. Figure 3a presents findings for Chip D, which had the thickest film after four rounds of deposition. Figure 3b presents the three-dimensional thickness profile of the SiO_2_ film on Chip D, measured using the Nexview NX2 white light interferometer (Zygo Corporation, Middlefield, CT, USA), which enables quick, large-scale measurements, as opposed to the time-consuming and inconvenient point-by-point manual measurement method of using a profilometer. The variation in thickness along the central radial direction, denoted by the white dashed line in Figure 3a, was determined from the three-dimensional profile. The results for Chips A, B, C, and D are summarized in Figure 3c.

The deposition times for Chips B, C, and D were 2, 3, and 4 times that for Chip A, respectively. The overall thickness of the whole chip was proportional to the deposition time (Figure 3c). A close examination of the third peak in Figure 3c, located at a radial distance of 130 mm, revealed that the films on Chips A, B, C, and D had thicknesses of 1675, 3381, 5054, and 6762 Å, respectively. Evidently, the thicknesses of the films on the chips were proportional to the total deposition time. Except for the first peak in Figure 3c, the other peaks and valleys corresponded well with the radial positions of the maximum and minimum arc angles indicated by the sinusoidal shadow mask design shown in Figure 1d. The positions of the peaks (at radial distances of 50, 90, and 130 mm) and valleys (at radial distances of 70 and 110 mm) suggest a period of 40 mm, consistent with the initial mask design. According to the design principle, the film thickness at the radial position r is proportional to the deposition time (or the opening arc angle). In our design, the arc angles at the radial distances of 90 and 130 mm were 80°; thus, the film should theoretically have the same maximum thickness at these two positions. Additionally, at the radial distances of 70 and 110 mm, the arc angles were both 36°, and the film should theoretically also have the same minimum thickness at these positions. However, thickness measurements indicated that the thicknesses of Chips A, B, C, and D increased gradually the with radial distance, which ran counter to the expectation that the same thicknesses would be obtained at the peak or valley positions. This inconsistency between theory and observation may be due to the uneven radial distribution of sputtering target molecules inside the chamber, although the underlying cause requires further investigation. Nevertheless, the thickness distribution across the four Si chips placed at the same radial positions but different circumferential positions on the carrier plate suggests that the proposed method is suitable for batch production.

### 3.3. Simulation and Experimental Demonstration of Linear Variable Filters

**Figure 4:**
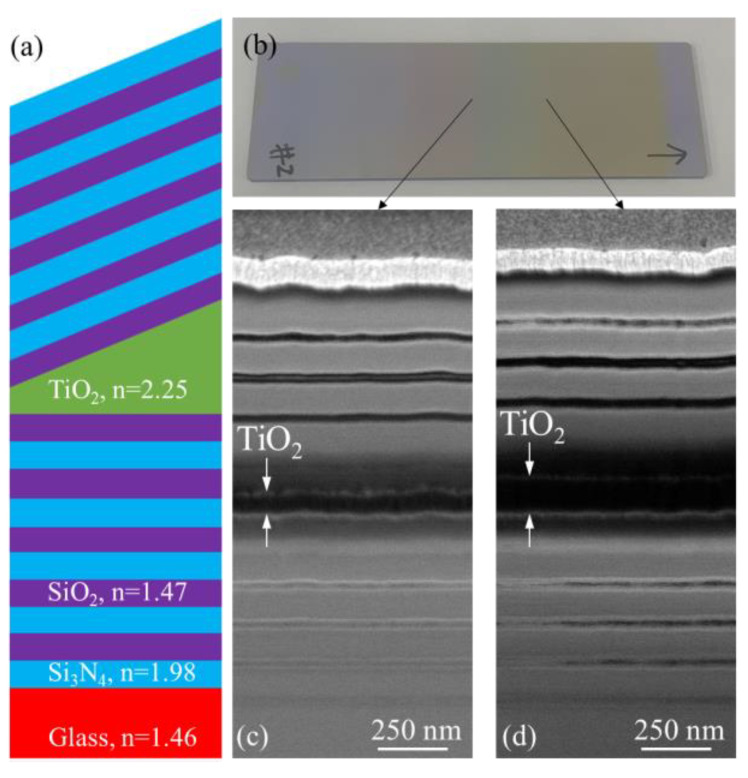
(**a**) Design of the LVF. (**b**) Image of the LVF. (**c**,**d**) Cross-sectional scanning electron microscopy images at two different locations, revealing different thicknesses of TiO_2_ films.

LVFs are useful in various applications, including multispectral and hyperspectral imaging systems [16]. Wan et al. designed an LVF-based platform for detecting fluorescence and determining absorption spectra [2]. In this study, an LVF based on a Fabry–Perot resonator was designed and fabricated to further verify the capability of the proposed method. The design of the LVF consisted of two sets of Bragg reflectors and a cavity layer with a linear thickness gradient (Figure 4a). The LVF was designed with a center wavelength of 600 nm. The top and bottom Bragg reflectors were composed of five pairs of alternating thin films of Si_3_N_4_ and SiO_2_, each with an optical thickness of a quarter wavelength. For the sake of the convenience of coating and to effectively evaluate the validity of a single cavity layer with a thickness gradient, the reflectors were uniformly deposited with 20 layers of constant thickness instead of a thickness gradient.

The first step in the manufacture of the LVF was the fabrication of the bottom Bragg reflector on the glass chips. Subsequently, two glass chips were placed on the carrier plate, and the shadow mask (as shown in Figure 1c) was used to deposit a TiO_2_ film with a linear thickness gradient. The deposition parameters are identical to those used in the Ni film deposition. The manufacture of the LVF concluded with the fabrication of the top Bragg reflector. Figure 4b shows an image of an LVF chip. Figure 4c,d display cross-sectional scanning electron microscopy images at two different locations on the chip. The dark and gray regions represent the Si_3_N_4_ and SiO_2_ layers, respectively. The SiO_2_ and Si_3_N_4_ layers at the top and bottom of the TiO_2_ film became difficult to distinguish from the TiO_2_ film because of the loss of contrast. Nevertheless, they still exhibited a clear contrast with the TiO_2_ layer, distinctly indicating different TiO_2_ film thicknesses (approximately 80 and 140 nm) at the two locations.

**Figure 5:**
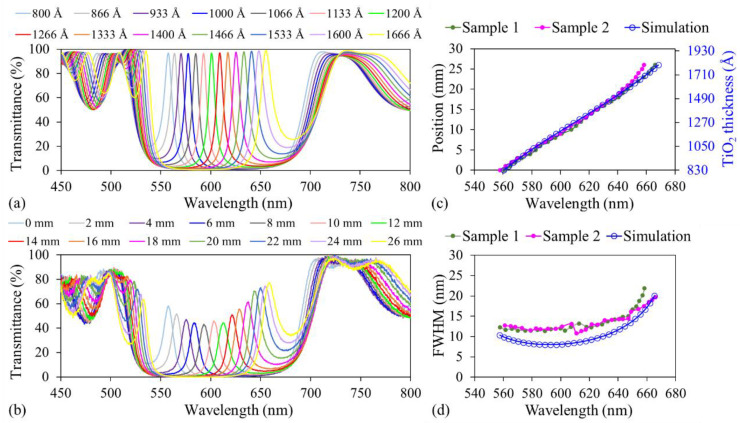
(**a**) Simulated transmission spectra for different thicknesses of TiO_2_ films. (**b**) Measured transmission spectra along the LVF. (**c**) LVF positions and the corresponding measured transmitted wavelengths, and the simulated transmitted wavelength and corresponding thicknesses of TiO_2_ films. (**d**) Comparison of FWHM derived from experimental and simulation results.

Because of the limited quality of scanning electron microscopy images and slight nonuniformities in the thickness of each Si_3_N_4_ and SiO_2_ layer, the approximate measured thicknesses of Si_3_N_4_ (130 nm) and SiO_2_ (55 nm) and their refractive indices, as measured by ellipsometry, were fed as inputs into the DiffractMOD 3.3 simulation tool (RSoft Design Group, Inc., Ossining, NY, USA). The refractive indices of the Si_3_N_4_ and SiO_2_ layers were 1.98 and 1.47, respectively. The simulation indicated that, when the TiO_2_ film thickness increased from 80 to 166.6 nm, the transmitted wavelength shifted from 557.5 to 655.4 nm (Figure 5a). A simple transmission setup was used to measure the transmission spectra along the LVF. A broadband light source (HL-2000-HP, Ocean Optics, Orlando, FL, USA) was coupled to a fiber (core diameter 400 μm) with a collimator at its exit. The light transmitted through the LVF was collected by another collimator attached to another fiber (core diameter 600 μm) that was connected to a spectrometer (USB2000+VIS-NIR-ES, Ocean Optics). The LVF was moved, and the transmission spectrum was measured as a function of the position of the LVF. Figure 5b indicates that the wavelength of transmitted light shifts from 558.16 to 660.33 nm within a distance of 26 mm.

Figure 5c illustrates the relationship between the measured transmitted wavelength and the relative LVF position for both samples, which were represented using green and pink markers. Figure 5c also presents the relationship between the transmitted wavelength and the thickness of the TiO_2_ film as discerned from the simulation results. The three curves in Figure 5c exhibit a good degree of overlap, which indicates that the thickness of the deposited TiO_2_ film indeed changes linearly along the LVF chip. Furthermore, the performances of both LVFs were nearly identical, further validating the potential of this method for batch processing. According to the simulation results in Figure 5c, the transmission wavelength shifted from 560.5 to 644.7 nm when the TiO_2_ thickness increased from 88.3 to 156.6 nm, which aligns with the experimental results from both Sample 1 and Sample 2 within a span of 20 mm. From these values, the thickness gradient was deduced to be approximately 3.415 nm/mm.

The LVFs exhibited reduced transmittance compared with the transmittance predicted by the simulation results, possibly because of the inconsistent thickness of the alternating layers in the Bragg reflector (Figure 4c,d). For both of the samples, the full width at half maximum (FWHM) varied between 11.4 and 21.9 nm and between 10.84 and 21.9 nm in the wavelength ranges of 557.81–658.3 nm and 560.98–661 nm, respectively (Figure 5b,d). By contrast, the FWHM varied from 8.0 to 20.01 nm, as calculated from the simulation results. The slightly broader FWHM obtained from the experimental results may primarily be attributed to the inconsistent thickness of each SiO_2_ and Si_3_N_4_ layer. In addition, each simulated transmission spectrum corresponds to a specific cavity thickness. In the experimental measurements, however, the diameter of the light spot on the LVF chip was approximately 1 cm. The transmitted light collected by the collimator consists of light coming from different TiO_2_ thicknesses, resulting in the broadening of the FWHM values in the measured transmission spectrum. A smaller illumination spot during measurement would result in increased transmittance and reduced FWHM values [15]. In summary, the relationships shared by the transmitted wavelength with the relative LVF position (Figure 5c) and FWHM (Figure 5d) for both samples indicate a consistent film deposition and filtering performance across the two samples.

## 4. Conclusions

This study proposed a new technique for depositing thin films with specific thickness profiles using a shadow mask with specially designed opening curves. The relationships between the opening curves of the shadow mask and the corresponding deposited thickness profiles were derived in detail, and two types of shadow masks were manufactured for the deposition of films with linear and sinusoidal thickness profiles. Linear thickness gradients could be controlled by simply adjusting the deposition time; thickness gradients of 49.3 and 86.8 Å/mm were achieved using the shadow mask. Additionally, the opening curve of a shadow mask was designed on the basis of the derived formulae to successfully deposit films with a sinusoidal thickness profile and a period of 40 mm.

To validate the practicality of the proposed method for manufacturing optical components, an LVF was fabricated that contained a cavity layer with a linear thickness gradient sandwiched between two Bragg mirrors. The LVF exhibited a linear shift in the transmitted wavelength as predicted by the simulation results; the transmitted wavelengths ranged from approximately 560 to 660 nm. These experiments demonstrated the ability of the deposition method to successfully produce films with linear thickness gradients, as well as the practical applications of the manufactured films as optical components.

The apparatus discussed in this study is simple and does not involve additional rotational or translational devices inside the sputtering chamber. The shadow mask and carrier plate were concentrically aligned, and the carrier plate was allowed to rotate during the deposition process. The experimental results not only demonstrated the designability of this approach but also its feasibility for the batch production of films with specific thickness profiles. Additionally, the rotation of the carrier plate reduces the effect of the nonuniform distribution of target molecules within the chamber, allowing the films deposited at the same radial distance but at different circumferential positions to achieve consistent thicknesses. This feature makes this method well-suited for mass production.

## 5. Patents

The United States Patent No.: US 11,499,219 B2 has resulted from the work reported in this manuscript.

## Data Availability

The data presented in this study are available on request from the corresponding author.

## References

[1] Emadi A., Grabarnik S., Wu H.W., de Graaf G., Hedsten K., Enoksson P., Correia J.H., Wolffenbuttel R.F. Spectral measurement using IC-compatible linear variable optical filter. Proceedings of the Conference on Micro-Optics 2010.

[2] Wan Y.H., Carlson J.A., Kesler B.A., Peng W., Su P., Al-Mulla S.A., Lim S.J., Smith A.M., Dallesasse J.M., Cunningham B.T. (2016). Compact characterization of liquid absorption and emission spectra using linear variable filters integrated with a CMOS imaging camera. Sci. Rep..

[3] Renhorn I.G.E., Bergström D., Hedborg J., Letalick D., Möller S. (2016). High spatial resolution hyperspectral camera based on a linear variable filter. Opt. Eng..

[4] Qian L.Y., Zhang D.W., Tao C.X., Hong R.J., Zhuang S.L. (2016). Tunable guided-mode resonant filter with wedged waveguide layer fabricated by masked ion beam etching. Opt. Lett..

[5] Yang J.-M., Yang N.-Z., Chen C.-H., Huang C.-S. (2021). Gradient waveguide thickness guided-mode resonance biosensor. Sensors.

[6] Ganesh N., Xiang A., Beltran N.B., Dobbs D.W., Cunningham B.T. (2007). Compact wavelength detection system incorporating a guided-mode resonance filter. Appl. Phys. Lett..

[7] Mika A.M. Linear-Wedge Spectrometer. Proceedings of the Conf on Imaging Spectroscopy of the Terrestrial Environment.

[8] Owen T.R. (1977). Spectrophotometer with Photodiode Array. U.S. Patent.

[9] Dami M., De Vidi R., Aroldi G., Belli F., Chicarella L., Piegari A., Sytchkova A., Bulir J., Lemarquis F., Lequime M. Ultra compact spectrometer using linear variable filters. Proceedings of the International Conference on Space Optics—ICSO 2010.

[10] Wang S.W., Liu D., Lin B., Chen X., Lu W. (2006). 16 × 1 integrated filter array in the MIR region prepared by using a combinatorial etching technique. Appl. Phys. B-Lasers Opt..

[11] Wang S.W., Li M., Xia C.S., Wang H.Q., Chen X.S., Lu W. (2007). 128 channels of integrated filter array rapidly fabricated by using the combinatorial deposition technique. Appl. Phys. B-Lasers Opt..

[12] Piegari A., Sytchkova A.K., Bulir J., Harnisch B., Wuttig A. Thin-film filters for a high resolution miniaturised spectrometer. Proceedings of the Conference on Advances in Optical Thin Films III.

[13] Abel-Tiberini L., Lemarquis F., Lequime M. (2008). Masking mechanisms applied to thin-film coatings for the manufacturing of linear variable filters for two-dimensional array detectors. Appl. Opt..

[14] Emadi A., Wu H., Grabarnik S., De Graaf G., Wolffenbuttel R. (2009). IC-compatible fabrication of linear variable optical filters for microspectrometer. Procedia Chem..

[15] Tang H.L., Gao J.S., Zhang J., Wang X.Y., Fu X.H. (2018). Preparation and Spectrum Characterization of a High Quality Linear Variable Filter. Coatings.

[16] Gao L., Smith R.T. (2015). Optical hyperspectral imaging in microscopy and spectroscopy—A review of data acquisition. J. Biophotonics.

